# Photosensitive Melanopsin-Containing Retinal Ganglion Cells in Health and Disease: Implications for Circadian Rhythms

**DOI:** 10.3390/ijms20133164

**Published:** 2019-06-28

**Authors:** Pedro Lax, Isabel Ortuño-Lizarán, Victoria Maneu, Manuel Vidal-Sanz, Nicolás Cuenca

**Affiliations:** 1 Department of Physiology, Genetics and Microbiology, University of Alicante, 03690 Alicante, Spain; 2 Department of Optics, Pharmacology and Anatomy, University of Alicante, 03690 Alicante, Spain; 3 Department of Ophthalmology, University of Murcia, 30120 Murcia, Spain; 4 Multidisciplinary Institute for Environmental Studies “Ramon Margalef”, University of Alicante, 03690 Alicante, Spain

**Keywords:** ipRGCs, circadian rhythms, aging, retinitis pigmentosa, P23H, Parkinson disease

## Abstract

Melanopsin-containing retinal ganglion cells (mRGCs) represent a third class of retinal photoreceptors involved in regulating the pupillary light reflex and circadian photoentrainment, among other things. The functional integrity of the circadian system and melanopsin cells is an essential component of well-being and health, being both impaired in aging and disease. Here we review evidence of melanopsin-expressing cell alterations in aging and neurodegenerative diseases and their correlation with the development of circadian rhythm disorders. In healthy humans, the average density of melanopsin-positive cells falls after age 70, accompanied by age-dependent atrophy of dendritic arborization. In addition to aging, inner and outer retinal diseases also involve progressive deterioration and loss of mRGCs that positively correlates with progressive alterations in circadian rhythms. Among others, mRGC number and plexus complexity are impaired in Parkinson’s disease patients; changes that may explain sleep and circadian rhythm disorders in this pathology. The key role of mRGCs in circadian photoentrainment and their loss in age and disease endorse the importance of eye care, even if vision is lost, to preserve melanopsin ganglion cells and their essential functions in the maintenance of an adequate quality of life.

## 1. Introduction

Intrinsically photosensitive retinal ganglion cells constitute a third class of photoreceptors within the retina, characterized by the expression of the photopigment melanopsin [1,2]. Globally, melanopsin-containing retinal ganglion cells (mRGCs) represent only between 0.3% and 0.8% of the total ganglion cells of the retina, but their roles are diverse and crucial and range from participating in image-forming vision [3,4,5,6] to other non-image forming functions such as regulation of circadian rhythms or activation of the pupillary light reflex. In the regulation of circadian rhythms, the light-induced activation of mRGCs travels through retinohypothalamic projections to the master circadian pacemaker, located in the suprachiasmatic nucleolus of the hypothalamus [2,7]. On a smaller scale, mRGCs project to the olivary pretectal nucleus regulating the pupillary light reflex [8,9,10,11]. In this sense, the existence of a close relationship between the circadian system robustness and the pupillary reflex response has been demonstrated [12]. Otherwise, a population of mRGCs project to brain regions involved in regulating mood and cognitive functions such as learning or memory [13,14,15]. Interestingly, not all melanopsin-containing cells project outside the retina, and some studies have proven the existence of melanopsin interneurons in the mammalian retina [16].

The functional integrity of the circadian system, partially dependent on melanopsin cells integrity, is an essential component of well-being and health, and its abnormal function may involve sleep disorders, cardiovascular problems, emotional disorders, or other pathological states [17,18,19,20,21]. Alterations in circadian rhythms have been reported in both aging [22,23,24,25] and disease, including ocular pathologies and blindness [26,27,28,29,30]. Aging has been associated with desynchronization and decreased circadian rhythm amplitude, which produces a gradual reduction of the nocturnal secretion of melatonin and variations in the sleep/wake phases [31,32]. These alterations have been primarily linked to a variety of pathologies associated with aging [33] rather than with aging itself. Nevertheless, circadian rhythm impairment described in aging and disease could be caused, at least in part, by morphological or functional changes of retinal ganglion cells and, more specifically, of melanopsin-containing ganglion cells [34,35,36,37].

Circadian clock disruption, generally accompanied by sleep-wake cycle abnormalities, may not only affect the life quality of patients but also trigger or accelerate the pathology progression in neurodegenerative diseases. In Alzheimer’s, Parkinson’s, and Huntington’s diseases, circadian rhythm alterations have traditionally been considered as natural consequences of neurodegenerative disorders [38,39,40,41,42], but they might actually contribute and worsen the neurodegenerative process [43,44].

The aim of this study is to review the evidence of melanopsin cell alterations associated with aging and neurodegenerative diseases and to correlate them with circadian rhythm disorders. The studies analyzed suggest that aging, ocular pathology, and neurodegenerative diseases induce retinal remodeling and loss of melanopsin-containing ganglion cells that correlates with the appearance of circadian disorders. This retinal degenerative process continues after the loss of cones and rods. Therefore, it is crucial to take care of the retina throughout life, even after having completely lost sight, to assure the preservation of melanopsin cells.

## 2. Melanopsin-Containing Ganglion Cells in Rodents and Humans

**Figure 1:**
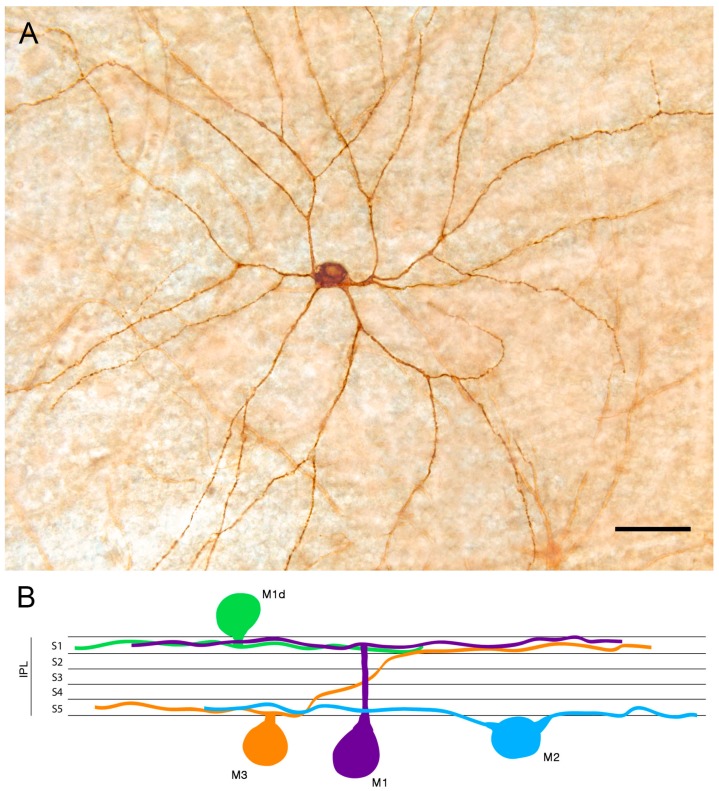
Melanopsin-containing ganglion cells (mRGCs) detected by conventional immunostaining: (**A**) Immunostaining of a displaced M1 cell (M1d) mRGC found in wholemount human retinas. (**B**) Diagram showing the structure of the mRGC types depending on their soma location and dendrite stratification in the inner plexiform layer (IPL) S1 or S5. Scale bar: 50 μm. (Modified from Ortuño-Lizaran et al., 2018) [42].

Melanopsin-containing ganglion cells are distributed throughout the inner nuclear and ganglion cell layers of the retina [45,46,47]. They can be classified into different cell subtypes depending on the location of their dendritic arborization within the inner plexiform layer (IPL), their retinal connections, and light responses [48,49,50]. According to morphological and physiological features, six types of melanopsin ganglion cells (M1 to M6) have been identified [6,50,51,52,53,54,55,56,57,58]. Three of these cell classes (M1, M2, and M3) have been described using traditional immunohistochemistry methods [37,42,59,60,61,62,63], while M4, M5, and M6 types express levels of melanopsin that are too low to be reliably detected by conventional immunostaining. These last types have been mainly detected by using signal-amplification immunolabeling methods or mouse reporter lines expressing fluorescent proteins under specific promoters. M1 cell dendrites stratify exclusively in the outer margin of the IPL (stratum S1), M2 cells project their dendrites to the inner margin of the IPL (stratum S5), and the less abundant M3 cells stratify in both the outer and inner margins of the IPL (S1 and S5, respectively). Nonetheless, the inclusion of M3 cells as a distinct melanopsin cell type remains controversial due to their lower density and their morphological and functional similarities with M2-type cells, so that the bistratified population of melanopsin cells has been suggested not to be an independent cell type [51,64,65]. Most subtypes of melanopsin-positive cells have the cell body in the ganglion cell layer (GCL) of the retina, but a population of M1 cells has the soma located in the inner nuclear layer (INL) [66]. These cells are called displaced M1 cells (M1d). The typical morphology of an M1d cell and a schematic representation of mRGC types revealed by conventional immunostaining are shown in Figure 1.

Using signal-amplification methods in immunohistochemistry protocols, researchers have been able to identify M4 cells in the retina of rodents and humans [6,52,56], which morphologically resemble the previously identified population of ON alpha RGCs [51,52,53,67]. The structure of M4 cells is similar to that of M2 cells, having dendrites exclusively in the inner margin of the IPL, but they are distinguished by the size and complexity of their dendritic fields and their large soma [6]. Besides, M2 dendrites stratify closer to the GCL than M4 dendrites [55]. Using the same techniques, new cells with morphological features similar to M1- and dM1-type cells but with an extremely large soma have been identified in human retinas and named gigantic M1 and gigantic displaced M1 cells, respectively [56]. This last cell type is abundant in the retinal periphery and may correspond to the rodents melanopsin interneurons that project intraretinally [16,56]. Additionally, M5 and M6 cells have been identified [50,57,58]. These cells are marginally immunoreactive for melanopsin, have relatively weak melanopsin-dependent light responses, and have not yet been identified in humans. M5 dendrites monostratify at the inner margin of the IPL [57], whereas the M6 cells’ dendritic arbor is bistratified [58].

**Figure 2:**
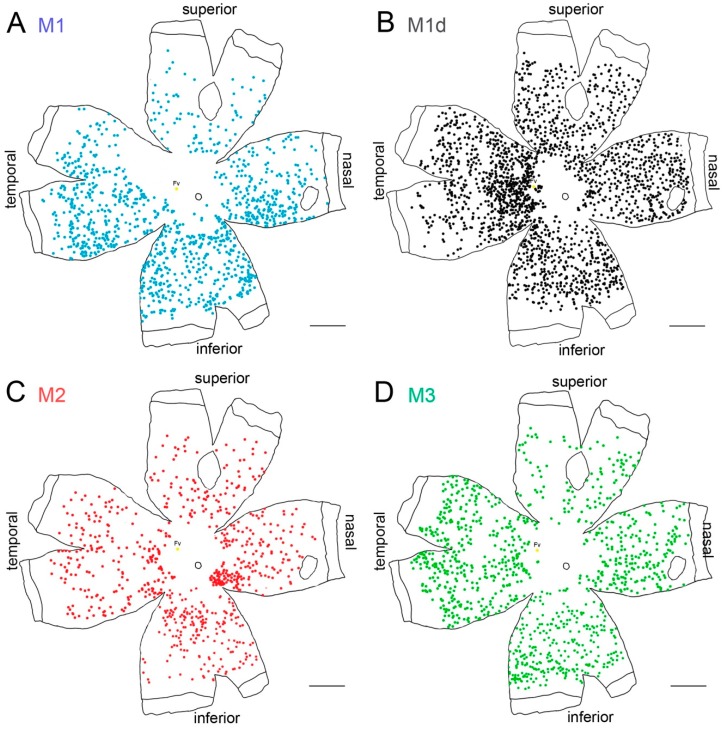
Melanopsin-containing ganglion cells on the human retina: (**A**–**D**) Representative drawings of a 56-year-old wholemount human retina showing the location of immunostained individual melanopsin-positive cells of different types. Each dot represents one mRGC and the color code indicates different mRGC types. Scale bar: 5 mm. (Modified from Esquiva et al., 2017) [37].

Melanopsin-expressing cells appear distributed throughout the entire rodent retina, even though a slightly higher density of melanopsin-expressing cells has been observed in the upper-temporal part of the rat retina [61,62,63]. In these animals, the number of M1 cells is higher than that of M2 and M3 cells, and dM1 cells represent a small group of mRGCs. Conversely, dM1 cells have been demonstrated to be the predominant cell subtype in human retinas [3,37,42,65,68]. Melanopsin cells are widely distributed throughout the entire human retina, even though a higher density has been observed in the perifoveal area, and a decreased number of these cells has been found in the vicinity of the optic nerve and in the peripheral retina [37,56,64,65] (Figure 2). Their morphology also varies with their location: close to the fovea, where the densities are higher, the dendritic arborization size is the smallest and in the periphery, where the density is lower, the dendritic size is greater [56,64,65].

## 3. Melanopsin-Containing Ganglion Cells in Aging

**Figure 3:**
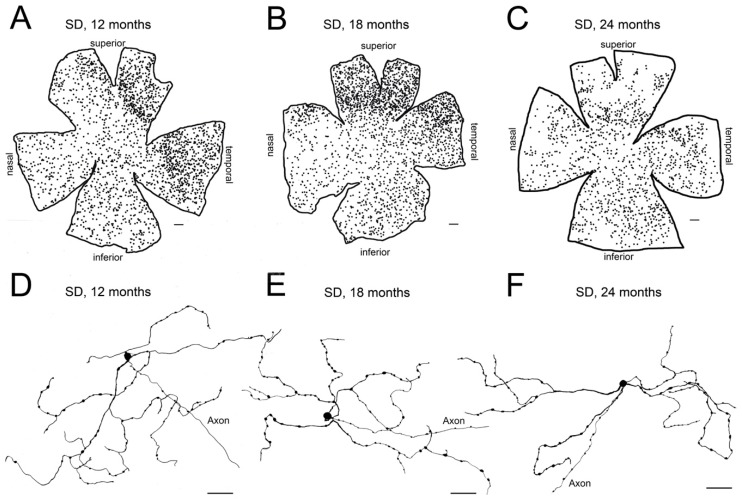
Age-related changes of melanopsin ganglion cells in control Sprague–Dawley rat retinas: (**A**–**C**) Representative drawings of wholemount retinas from Sprague–Dawley (SD) rats at 12 (**A**), 18 (**B**), and 24 (**C**) months-of-age. (**D**–**F**) Representative drawings of the soma and complete dendritic field of mRGCs from a region of the central retina (between the superior and nasal quadrants) of Sprague–Dawley rats at 12 (**D**), 18 (**E**), and 24 (**F**) months-of-age. Drawings were made using a camera lucida and reveal immunostained mRGCs. Scale bar: **A**–**C**, 500 μm; **D**–**F**, 50 μm. (Modified from Lax et al., 2016) [63].

Vision decrease with age [69,70], and aging has been associated with qualitative and quantitative changes in retinal neurons [71,72,73,74,75]. In this context, electrophysiological and psychophysical methods for retinal function testing show a loss of visual function throughout age in healthy rats, with progressive decay in electroretinographic responsiveness and visual acuity [76,77]. Aging has also been associated with alterations in circadian rhythms [22,23,24,78,79] that can be attributed, at least in part, to the mentioned visual loss. Related to this fact, it has been demonstrated that melanopsin-positive cell density is maintained in normal rats at 12 and 18 months of age [36,61,63] but shows a decline in number at 24 months, being up to a 50% lower than that observed in 12- and 18-month-old control animals [63] (Figure 3). In agreement with this finding, it has been reported that body temperature and locomotor activity circadian rhythms are more robust in the young adult, as compared to elderly rats [63].

**Figure 4:**
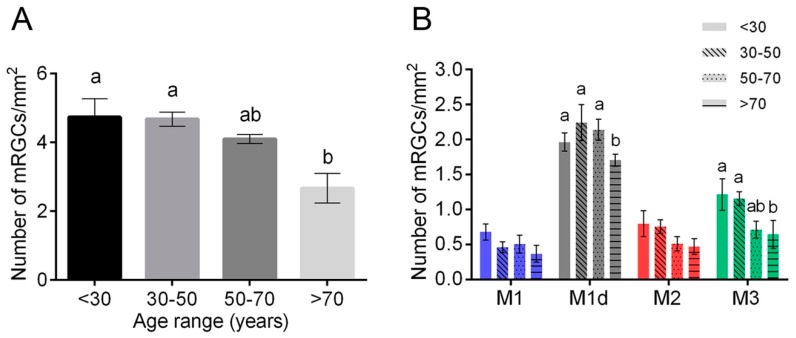
Age-related changes of melanopsin ganglion cells in human retinas: (**A**,**B**) Mean density of total mRGCs (**A**) and different types of mRGCs (**B**) in human retinas between 10 and 81 years-of-age. Different letters above the histograms indicate statistically significant differences between ages (*p* < 0.05). (Modified from Esquiva et al., 2017) [37].

In humans and non-human primates, older adults present reduced rhythm amplitudes and age-related rhythm fragmentation, indicating that circadian rhythms are altered with age [80]. However, some experiments show no age-related changes in pupil responses mediated by mRGCs [81], and that the retinohypothalamic pathway seems to be relatively unaffected by aging [82]. These results are in accordance with the relatively stable density of mRGCs over time, that is normally maintained in healthy subjects until the age of 70 [37]. From 50 years onwards, a tendency of decrease (about 13%) has been observed, but a deep fall in mRGC number (approximately a loss of a 44%) occurs after 70 years-of-age [37] (Figure 4). The characterization of morphological and dendritic parameters also indicates atrophy of mRGC dendritic arborization at late ages. From 50 years old onwards, mRGC plexuses decrease their complexity, and after 70 years-of-age dendritic trees show little overlapping and few contacts between the scarce number of remaining melanopsin-positive cells [37].

## 4. Melanopsin-Containing Ganglion Cells in Retinal Diseases

Ocular pathologies and blindness have been classically associated with circadian rhythm disturbances that depend on the degree to which the perception of light is affected [83,84,85,86]. Many studies correlate circadian disorders with inner retinal diseases, such as glaucoma [84,87,88], diabetic retinopathy [85,89], or retinal ischemia [86], diseases in which circadian rhythm alterations in these diseases have been experimentally related to the loss of mRGCs in the retina. Melanopsin-containing ganglion cells have shown a marked resistance to injury, showing more resistance to neurodegeneration than the rest of the ganglion cells of the retina [90,91,92,93,94,95]. As an example, the mRGC/RGC ratio in controls and patients with mild glaucoma represent about 0.3%, while in severe glaucoma the mRGC number represents 14% of the total ganglion cells of the retina [91]. Nevertheless, in spite of their resistance, many other studies have shown a loss and impairment of melanopsin cells associated with retinal disease. A loss of approximately 50% of mRGCs has been described in rodents with experimental glaucoma [96], and a decrease in mRGCs has been described in severe staged glaucoma patients [97]. In animal models of diabetic retinopathy, retinal degeneration also results in a loss of mRGCs (about 75% less) [98].

**Figure 5:**
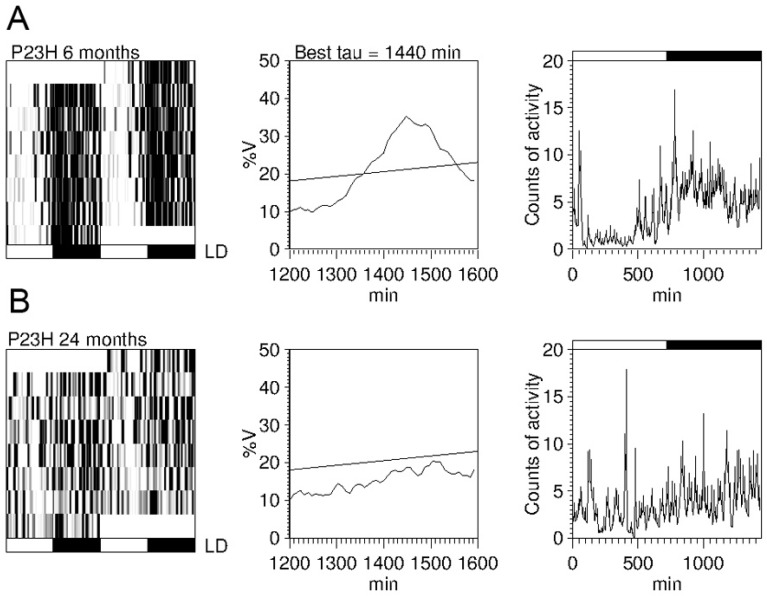
Circadian rhythms of locomotor activity in P23H line 1 rats: (**A**,**B**) Example of actograms (left panels), periodograms (middle panels), and mean waveforms (right panels) at the ages of 6 (**A**) and 24 (**B**) months for a P23H-1 rat exposed to a 12:12 LD cycle. All data were obtained from the same animal. Light and dark schedules are represented by white and dark bars, respectively. (Modified from Lax et al., 2016) [63].

Apart from inner retina diseases, circadian dysfunctions have also been reported in advanced stages of diseases affecting the outer retina, such as retinitis pigmentosa [18,27,28,99]. It has been demonstrated that retinal degeneration positively correlates with the occurrence of circadian dysfunctions in P23H line 3 rats [100], an animal model of retinitis pigmentosa (RP), and that advanced stages of the degenerative disease correlate with reduced rhythm amplitudes, weaker coupling strength of the rhythm to environmental zeitgebers, and higher rhythm fragmentation in P23H line 1 rats [63] (Figure 5). A more recent study demonstrated that administration of exogenous cannabinoids protects from circadian rhythmicity impairment and vision loss in P23H line 3 dystrophic rats [101].

**Figure 6:**
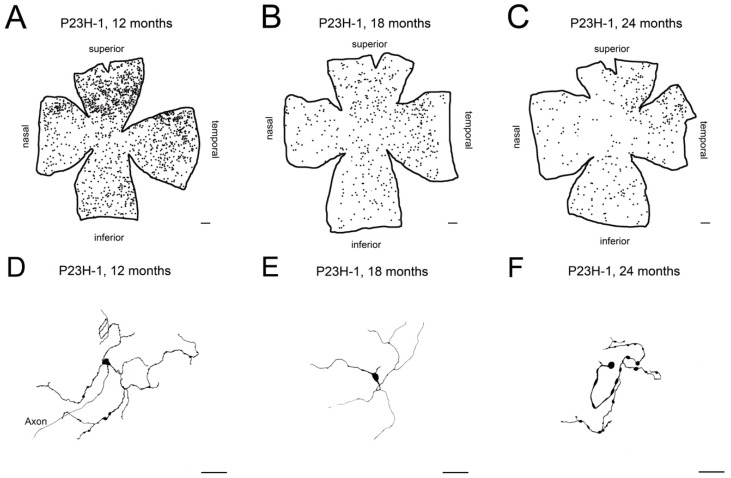
Age-related changes of melanopsin ganglion cells in P23H line 1 rat retinas: (**A**–**C**) Representative drawings of wholemount retinas from P23H-1 rats at 12 (**A**), 18 (**B**), and 24 (**C**) months-of-age. (**D–F**) Representative drawings of the soma and complete dendritic field of mRGCs from a region of the central retina (between the superior and nasal quadrants) of P23H-1 rats at 12 (**D**), 18 (**E**), and 24 (**F**) months-of-age. Scale bar: **A**–**C**, 500 μm; **D**–**F**, 50 μm. (Modified from Lax et al., 2016) [63].

Retinitis pigmentosa is characterized by a progressive loss of photoreceptors [102,103,104], accompanied by degenerative changes in the inner retina [105,106,107,108,109,110] and the subsequent degeneration of retinal ganglion cells [35,111]. Among others, retinitis pigmentosa is associated with a progressive degeneration of melanopsin-containing ganglion cells, whose density, integrity, and dendritic arborization are decreased in advanced stages of the disease [36,61] (Figure 6). Experimental evidence indicates that the progressive deterioration of melanopsin cells in advanced stages of retinitis pigmentosa positively correlates with progressive alterations in circadian rhythms [63].

The degenerative effects of retinitis pigmentosa on the number and morphology of melanopsin cells occurs relatively late compared to the degeneration observed in other retinal neurons. This relatively high resistance of melanopsin cells to cellular injuries has been attributed to both morphological and physiological properties, such as having a large soma, long and sparsely branching dendritic fields, and intrinsic light sensitivity [112,113,114,115]. This suggests that, even in severe cases of outer retinal diseases in humans, a functional population of melanopsin cells can still persist, and its care is crucial to maintain a better quality of life even if vision is lost. Accordingly, neuroprotective strategies to reduce melanopsin cell degeneration might play a decisive role in preventing sleep and circadian rhythm disorders associated with retinal degeneration.

## 5. Melanopsin-Containing Ganglion Cells in Neurodegenerative Diseases

**Figure 7:**
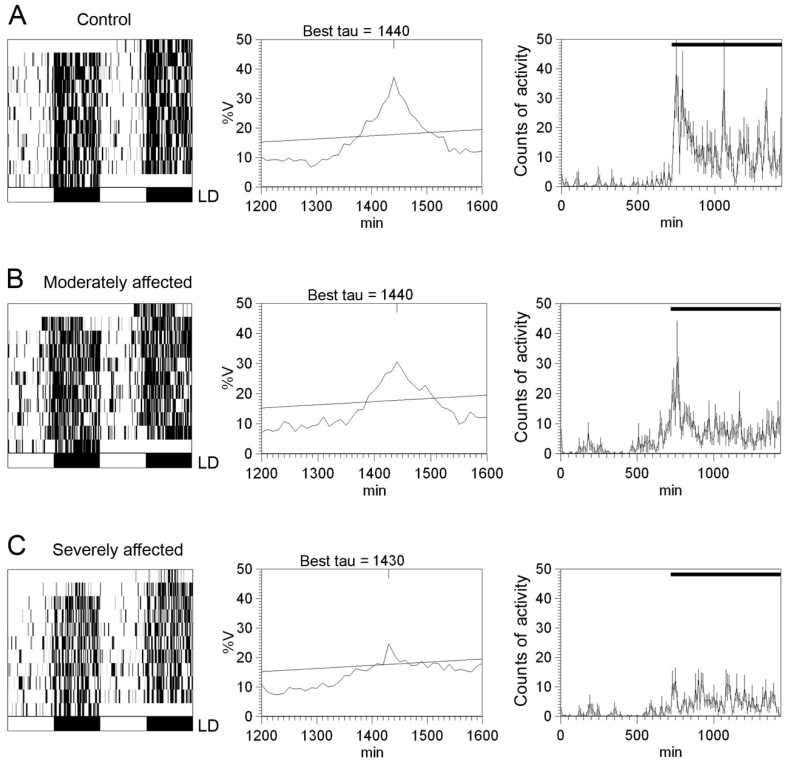
Circadian rhythms of locomotor activity in rotenone-induced Parkinsonian rats: (**A**–**C**) Representative locomotor activity actograms (**left** panels), periodograms (middle panels), and mean waveforms (**right** panels) for a control animal (**A**), an animal moderately affected by rotenone (**B**), and an animal severely affected by rotenone (**C**) exposed to a 12:12 LD cycle. Light and dark schedules are represented by white and dark bars, respectively. (Modified from Lax et al., 2012) [134].

In addition to aging and retinal diseases, circadian rhythms are also impaired in neurodegenerative diseases such as Alzheimer’s disease (AD), Parkinson’s disease (PD) and Huntington’s disease [21,43]. In Parkinson’s disease, numerous studies have shown that, apart from the disease-specific clinical motor features [116,117,118,119], patients also exhibit several non-motor symptoms including visual impairment [120,121,122,123], deterioration of the pupillary light reflex [124,125,126], and sleep disorders [116,117,127,128,129,130]. Similar circadian disturbances have been observed in various experimental PD models [131,132,133]. In the rotenone rat model of Parkinson’s disease, the circadian rhythm of both locomotor activity and body temperature showed decreased amplitudes and higher rhythm fragmentation when compared to control rats [134]. The magnitude of these circadian alterations positively correlates with the degree of motor impairment (Figure 7).

**Figure 8:**
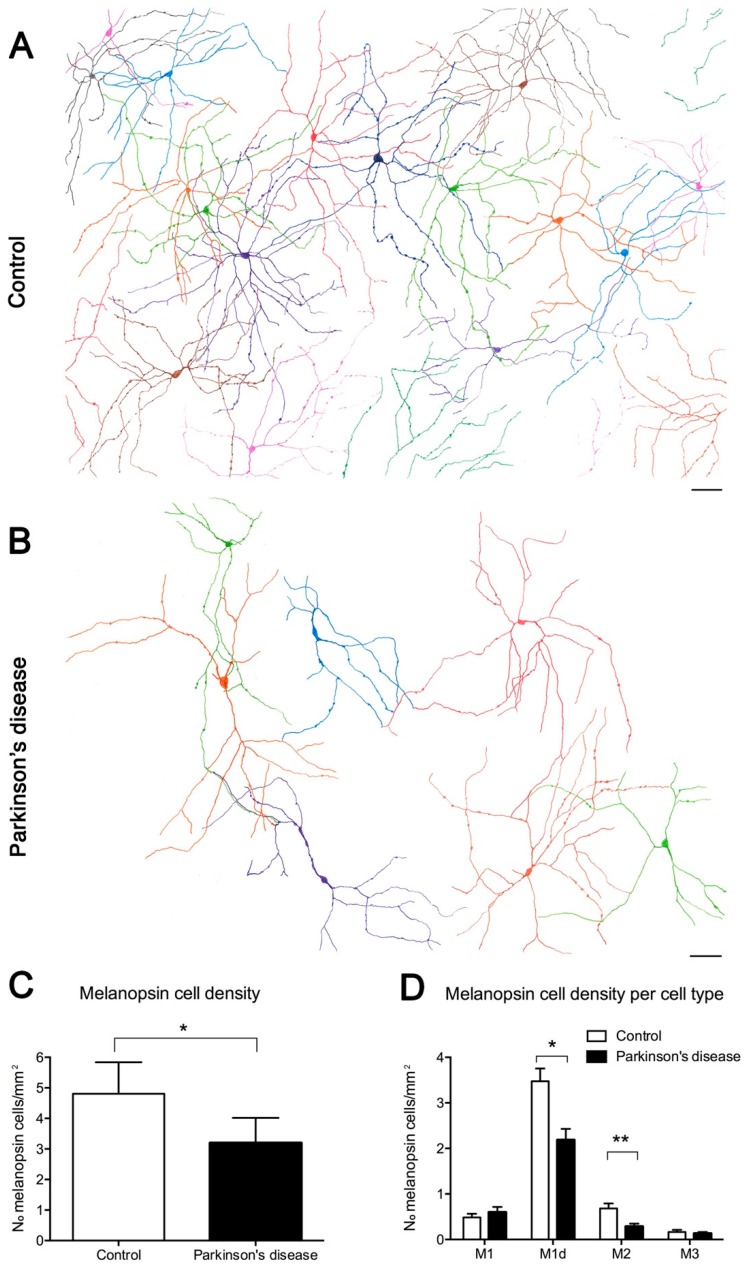
Melanopsin ganglion cells in Parkinson’s disease (PD): (**A**–**B**) Representative drawings of control and PD retinal fields. (**A**) Melanopsin plexus in a control wholemount retina. (**B**) Melanopsin plexus in a PD wholemount retina. Each color defines an individual mRGC. (**C**) Total mRGC quantification (number of mRGCs per mm^2^) and comparison between control and PD subjects. (**D**) Comparison of the mRGC density per cell type in control and PD subjects. Scale bar, 100 μm. (Modified from Ortuño-Lizarán et al., 2018) [42].

Numerous studies have shown that the retina is also affected in Parkinson’s disease [135]. Degeneration of photoreceptors and impairment of the dopaminergic system have been shown in animal models of PD [136], and immunohistochemical studies have revealed the presence of phosphorylated-α-synuclein immunoreactive neuronal elements in postmortem retinas of PD patients [137], where its density significantly correlated with synucleinopathy density in the brain of the same PD patients [123]. On the other hand, the pupil light-reflex deterioration in PD patients may indicate that melanopsin-mediated retinal inputs are impaired [126]. In fact, morphological and numerical analysis of mRGCs in PD patients has demonstrated that the retinal melanopsin system is impaired in the disease [42]. This study shows a reduction in the number of mRGCs in PD patients, accompanied by a drastic reduction in their plexus complexity and by morphological alterations like decreased Sholl area, fewer ramifications and terminal points [42] (Figure 8). On the other hand, previous studies have demonstrated that dopaminergic neurons of the retina make synapses with mRGCs [64,138,139], and functional modulation of mRGCs by dopamine has been reported [138,139,140,141]. These studies suggest that circadian dysfunction in PD pathology might be partially attributable to altered dopaminergic modulation of melanopsin cells.

Alzheimer’s disease patients also suffer from circadian rhythm dysfunction and reduction of sleep efficiency. Immunohistochemical analysis of retinal sections of AD and controls revealed a loss of mRGCs in the disease, compared to controls. In addition, cell morphology was affected and mRGCs seemed to have smaller soma and thinner dendrites in AD [142]. Degeneration of melanopsin ganglion cells in AD may explain, as in PD, the circadian rhythm impairment described in patients [142,143,144]. Although a relationship between circadian rhythm dysfunction and mRGC loss has been only described until now in AD and PD, it has been proved that the retina is affected in many brain-predominant neurodegenerative diseases and it may be a useful tissue to study the neurodegeneration subjacent to circadian impairments in other pathologies.

## 6. Conclusions

Overall, this review reports evidence that both the number and structure of melanopsin-positive cells are affected by aging, retinal disease, and neurodegenerative disorders, and that these alterations correlate with the appearance of circadian rhythm disorders. Melanopsin ganglion cells show more resistance to cell injury than the rest of the ganglion cells of the retina, presumably due to both morphological and physiological properties. However, these cells are equally affected in some neurodegenerative conditions, especially in advanced stages of the degenerative process. The correct functioning of the circadian system and the melanopsin cells constitutes an essential component of well-being and health. Accordingly, taking care of the retina to preserve melanopsin ganglion cells and their essential functions, even if vision is lost, is essential in the maintenance of an adequate quality of life. On the other hand, this review shows evidence that mRGCs may be affected in brain-predominant neurodegenerative diseases and that the study of the retina may be a key element to understand in detail the neurodegeneration underlying the circadian alterations observed in different pathologies.

## Abbreviations

mRGCs	Melanopsin-containing ganglion cells
IPL	Inner plexiform layer
GCL	Ganglion cell layer
INL	Inner nuclear layer
M1d	Displaced M1 cells
AD	Alzheimer’s disease
PD	Parkinson’s disease
